# Emergency Portacaval Shunts in Variceal Hemorrage: A Critique of Critics

**DOI:** 10.1155/1994/80230

**Published:** 1994

**Authors:** Harold O. Conn

**Affiliations:** Yale University School of Medicine, New Haven Connecticut and D.V.A. Medical Center West Haven Connecticut 06516 USA


### EMERGENCY PORTACAVAL SHUNTS IN VARICEAL HEMORRAGE: A CRITIQUE OF CRITICS

### ABSTRACT

*Orloff, M. J., Orloff, M. S., Rabotti, M. and Girard, B. (1992) Is portal-systemic shunt worthwhile in Child's Class C cirrhosis. Annals of Surgery, 216, 256–268.*

**A prospective evaluation was conducted of 94 unselected patients ("all comers") with biopsy-proven Child's class C cirrhosis (93% alcoholic) and endoscopically proven acutely bleeding esophageal varices who underwent emergency portacaval shunt (EPCS) (85% side-to-side, 15% end-to-side) within 8 hours of initial contact (mean, 6.1 hours) during the past 12 years. Follow-up has been 100% and includes all patients for at least 1 year, and 61 patients (65%) for 5 to 12 years. Incidence of serious risk factors on initial contact was: ascites, 97%; jaundice, 86%; portal-systemic encephalopathy including past history, 71%; severe muscle wasting, 96%; alcohol ingestion within 7 days, 66%; delirium tremens, 16%; serum albumin, ≤ 2.5 g/dL 76%; indocyanine green dye retention ≥ 50% in 45 minutes, 66%; serum glutamic-oxaloacetic transaminase ≥ 100 units/L, 60%; hyperdynamic cardiac output ≥ 6 L/minute, 98%. Mean Child's point score was 13.7 out of a maximum of 15. EPCS reduced mean corrected free portal pressure from 286 to 23 mm saline, and permanently controlled variceal bleeding in every patient. Of the 94 patients, 74 (80%) left the hospital alive and 68 (72%) survived 1 year. Five year actuarial survival rate is 64%. Hepatic failure was the main cause of death during initial hospitalization as well as during follow-up, when it was related to continued alcoholism. Portal-systemic encephalopathy, which was present on initial contact in 55% of patients, occurred at some time during follow-up in 18.7%, but was recurrent and required dietary protein restriction in only 9%, all of whom had resumed alcoholism. The low incidence of portal-systemic encephalopathy was attributable to the lifelong program of follow-up with regular dietary counseling and continued emphasis on both protein restriction to 60 g/day and abstinence from alcohol. Abstinence was sustained in 69%, liver function improved in 82%, general health was judged excellent or good in 73%, and Child's risk class converted to class B in 73% and class A in 21%. Excluding retirees because of age, 42% were gainfully employed or engaged in full-time house-keeping. Long-term shunt patency was documented in 100% of survivors by yearly angiography or Doppler ultrasonography. It is concluded that EPCS within 8 hours of initial contact permanently controls variceal hemorrhage and results in prolonged survival and a life of acceptable quality in many alcoholic cirrhotic patients in Child's class C. The improvement in our results compared with the previously reported results of all forms of treatment is attributable to rapid diagnosis, prompt operation, an organized system of care, and rigorous lifelong follow-up. The widespread pessimism about these patients is not justified, and the suggestion that liver transplantation offers the only or best hope is unwarranted.**

KEY WORDS: Portacaval shunt, Child' Class, portal hypertension, Cirrhosis, bleeding esophageal varices

### PAPER DISCUSSION

*Don't throw out the baby with the bath water Proverb (first citation) Keppler 1610*

I have long enjoyed the policy of the *American Journal of Surgery* which appends comments by the audience and the responses of the speaker about papers presented at their annual meeting. Often these discussions are as informative as the articles themselves. The discussion of Marshall Orloff's article on emergency portacaval shunts (EPCS) in patients with Child's C cirrhosis is a case in point[1]. Dr. Bernard Langer of Toronto, a highly respected surgeon, commented that Orloff's article is "···remarkable for its ··· low operative mortality and its long term survival···for the fact that ··· 94% of the Child's C patients reverted to Child's A or B, and that these figures ··· are at odds with almost anything else that has been published in the literature". Dr. Langer seems incredulous about these data and has difficulty in accepting them at face value. However, Dr. Langer, whom I have known for almost 30 years, is a true gentleman, and, in my opinion, his comments are as close as he can come to cry, "Fraud". Indeed, he didn't. Instead, he suggested other explanations for these egregious data. Could the delay of 24 or 48 hours be responsible for the difference in Orloff's results and those of others? Is it possible that Orloff's patients are healthier than true childs Class C patients, due to differences in the Child-Turcotte and Child-Turcotte-Campbell classifications? He mutes his disbelief in concluding that, "I am not convinced that surgery should be first-line therapy for every patient who is bleeding from varices". Others, who are not so gentle, who do not read so carefully, and/or who are irritated by Orloff's provocative style, seem to dismiss his results as exaggerations or worse, and to close their minds to his explanations of how he achieved such astounding results.

For over a quarter of a century Orloff has been the standard bearer for EPCS and he has made UCSD the world's capital of emrgency shunts. His message is loud, clear and strident. In a long series of articles he has explained why he believes that EPCS is the treatment of choice for bleeding varices and in the process contradicts the cumulative results of dozens of experienced investigators. In the latest article in this series, which can be considered the scripture on which this sermon is based, he and his associates present the observations that are so difficult for so many to accept. *What are these astounding results*? The article describes their findings in "...94 *consecutive, unselected*" patients with *Child's Class C cirrhosis* in whom this difficult procedure was *successfully accomplished in all within 8 hours of admission* to the hospital (mean 6.1 hours) and in whom the procedure *stopped the bleeding immediately* and "...permanenty *controlled the hemorrhage in every patient, none of whom was lost to follow-up* before 5 years". Furthermore, *80% left the hospital alive, 64% survived for 5 years, abstinance was sustained in 69%* of this largely alcoholic group, *in only 9% portal-systemic encephalopathy* (PSE) "...was recurrent and required dietary protein restriction..." , long term shunt patency was documented in 100% by yearly angiography or Doppler ultrasonography. These are outrageous claims, outrageously stated. The single most impressive statement is that *Child's class C* "...reverted to Class B in *73% and to Class A in 21%*". In vintage Orloffian style he ends with the zinger, "...*there is no justification for the widespread pessimism and fatalistic attitude* about patients with class C cirrhosis who bleed from varices". That is pouring salt into the wound! To my knowledge no one has reported as high rates of successful performance of EPCS, of follow-up, of abstension from alcohol, of survival, of shunt patency, of improvement in hepatic status or as low a rate of postshunt PSE.

*Why are these results so difficult to accept*? First, because they are so much better than any other reported data. Second, I believe, because they are presented in such a direct, cheerful, ingenuous manner that the reader is made to feel inadequate, demeaned and defensive. His confrontational style, which is probably subconscious, attracts attention. For one to state emphatically that one's work is superior to all others' rarely elicits a warm, friendly response. However, we must take care not to discard the substance because of the style.

Let's examine the individual claims: 94 consecutive anythings is pretty good, but 94 consecutive EPCS in child's class C patients represents a record that may never be broken. The absence of a single shunt occlusion is another world's record. Only 9% with chronic post-shunt PSE is truly noteworthy. I suspect that rigorous examination by experienced encephalopathologists might well identify additional cases, but I doubt many more. The detection of subtle PSE is a subjective art form[2]. Besides, the patients all had been on mild dietary protein restriction (60 g per pay). Actually, the portal decompression, which prevents hemorrhage from varices, which is one of the most common causes of PSE, may itself have diminished the frequency of PSE.

Let's put the post-shunt PSE in this investigation into proper perspective. More than half of these 94 patients had had PSE when they were first examined, while they were actively bleeding. An additional 16% gave a history of previous PSE; thus, 71% of these patients had apparently exhibited PSE prior to EPCS. Patients who develop PSE are unusually susceptible to subsequent episodes[3]. In the post-operative period 22 patients (23%) experienced PSE, usually in the presence of hypokalemic, Metabolic alkalosis, a known precipitant of PSE[4]. After EPCS 9 patients experienced a single episode of PSE. An additional 9 patients (7%) developed recurrent PSE that required further reduction of protein intake and the administration of lactulose. All 18 patients had resumed heavy alcohol intake despite repeated emphasis during follow-up about abstinence from alcohol and protein restriction. These associations demonstrate the multiplicity of factors that contribute to post-shunt PSE and emphasizes two of the cornerstones of Orloff's follow-up therapy—abstinence and protein restriction.

In an earlier controlled comparison of PSE in shunted and unshunted cirrhotic patients with esophageal varices the results were not dissimilar[5]. Recurrent PSE occurred in 20% of the shunted patients and in only 3% of the unshunted patients. The most common precipitant of PSE in the shunted patients was dietary protein and in unshunted patients it was gastrointestinal bleeding. Thus the presence of a patent PCS per se reduces the risk of PSE in shunted patients by reducing the risk of homorrhage.

I have had the opportunity to assess the validity of some aspects of Orloff's claims by interrogating physicians, surgeons, house officers and GI fellows at UCSD. I have not found a single instance in which any patients have been knowingly excluded from having EPCS. When I informed Orloff of this surveillance he volunteered to assist my agents. Probably, some patients who were thought to be especially poor operative risks were diverted to other hospitals, but it is not possible to estimate the extent of this referral bias.

Furthermore, as a member of the External Advisory Committee of the NIH sponsored San Diego EPCS vs Sclerotherapy randomized clinical trial, I have had the opportunity to examine the records of some of these patients. I have been unable to uncover a single episode of deception. I found the records from this investigation to be carefully documented, and amazingly complete.

To me the reversal of Child's C patients to A or B in the large majority of patients is the single most remarkable observation. This effect may largely reflect improvement in ascites, which can reduce Child's score by 1 or 2 points and may result in improved nutrition. Similar claims have been made for the effect of peritoneavenous shunts[6]. The second most surprising claim to me, is the statement that "...no patient with bleeding varices has ever refused EPCS at UCSD University Hospital". I have confronted Orloff directly about this statement:

"Would you dare refuse if I told you that the operation was essential to your wellbeing?" he responded, loudly, pushing his pugnacious jaw close to mine.

"No, sir," I replied.

Orloff believes that the key principles responsible for his success are (a) rapid diagnosis, (b) prompt surgery, (c) an organized system of care and (d) lifelong follow-up. I add (e) an excellent, experienced surgeon.

Postoperatively Orloff's patients are followed closely and each complication is promptly and vigorously treated Good-nutrit. Nutrition and abstinance are stressed. In my opinion the key to EPCS is the prompt performance of the shunt, which saves at a minimum 12 to 36 hours of potential clinical deterioration. An early emergency shunt diminishes the amount of blood lost and blood replacement. Continued bleeding induces vascular instability, a catabolic state and a decrease in oxygen delivery to all the vital organs. It can cause shock with severe renal and central nervous system injury. Encephalopathy, too, is a component of the child's score and prompt cessation of bleeding diminishes the increase in hyperammonemia and can thus reduce Child's score by 1 or 2 points. Each blood transfusion increases the hemolytic component and the opportunity for electrolyte abnormalities, under-or overhydration, azotemia, transfusion mismatches and the transmission of infectious diseases.

The shunt procedure itself eliminates the portal hypertension and improves the hepatic, splanchnic and systemic circulations. In addition, it decreases the amount of ascites which may improve systemic and renal hemodynamics and decrease the risks of spontaneous bacterial peritonitis and the hepatorenal syndrome.

Shunts performed within a few hours of the onset hemorrhage appear to be safer than those performed a few days or weeks later because the patient at that time is in as good health as he or she will ever again achieve and can tolerate the effects of trauma, anesthesia, blood loss, etc., better. Timing is very important. Child's score may not be the same 3 hours after admission as it is 24 hours later. The higher serum bilirubin concentration may easily add 1 or 2 points to the Child's score, changing a patient from class A to B or B to C.

Some of these concepts have been confirmed in the preliminary report of a RCT in which EPCS has been compared with emergency medical therapy (vasopressin and/or esophageal balloon tamponade) followed by elective PCS[8]. The 45 patients in the two groups were similar in demographic and clinical features and to the large series under discussion here. The outcome was greatly improved by EPCS, after which all patients stopped bleeding, compared to $< 50\%$ in the medical-elective PCS group. The mean reduction in transfusion requirements was 10 units, and survival was significantly better at 30 days, 1 year and 5 years. Furthermore, the mean cost of EPCS was no greater and after 30 days became progressively less. This important study, which has so far been reported only in abstract form, has now been submitted for publication.

Clearly it is essential to have an organized, experienced team in place. One needs an energetic endoscopist, an aggressive surgeon, a capable anesthesiologist and the rest of the supporting cast to undertake prompt diagnostic procedures, perform major surgery quickly, to provide nutritional support and to carry the patient through the post-operative period and a long term follow-up.

Despite all this praise, Orloff is not perfect. He makes the point that he is using the Child-Turcotte-Campbell system of grading the severity of cirrhosis[7], but he is not. In the Campbell modification it is necessary to determine whether the ascites is "easily controlled" or "difficult to control", decisions that require multiple observations. In Orloff's series ascites is graded on its preserved absence and volume immediately after a discussion. Furthermore, there are better methods of treating active bleeding than by infusing vasopressin.

One aspect of these data must be taken into serious consideration, i.e. "*The Wizard Phenomenon*". A wizard is defined as "one endowed with exceptional skill...and able to achieve something held to be impossible"[9]. Orloff's reported feats with EPCS surgery easily satisfy this definition. One must worry whether subsequent surgeons can equal Orloff's achievements, but this lis the way of the world. In all fields of human endeavor progress is made by incremental breakthroughs. On October 18, 1968, for example Bob Beamon made a prodigious longjump at the Olympics is Mexico City (8.90 m), almost 22 inches, longer than the existing world record, which had changed less than 8 inches in 33 years. In 1991, this new record, too, was broken[10]. Starzl *et al.*, performed the first liver transplant in a human in 1963 a unique occurrence thought then to be of little practical value, and now, thirty years later, this procedure is performed as standard therapy at hundreds of hospitals. EPCS has been performed by many surgeons for many years, perhaps, not so successfully as Orloff, but I predict that using a different time frame, surgeons will be able to perform this procedure as rapidly and effectively as the Wizard of San Diego.

After considering all these data I think that EPCS may be the one true treatment of actively bleeding varices and that Orloff is its prophet.

*References*

1. Orloff, M. J., Orloff, M. S., Rambotti, M. and Girard, B. (1992) Is portal-systemic shunt worthwhile in Child's Class C cirrhosis? *Ann. Surg.*, **216**, 256–268.
2. Conn, H. O. (1994) Subclinical hepatic encephalopathy. In Hepatic Encephalopathy: Syndromes and Therapies, Ed. Conn, H. O. and Bircher, J. Medi-Ed Press, East Lansing, Michigan, p. 27–42.
3. Zieve, L., Doizaki, W. M. and Zieve, F. J. (1974) Synergism between mercaptans and ammonia or fatty acids in the production of coma: a possible role for mercaptans in the pathogenesis of hepatic coma. *J. Lab. Clin. Med.*, **83**, 16–28.
4. Conn, H. O. and Lieberthal, M. M. (1979) The Hepatic Coma Syndromes and Lactulose, Williams & Wilkins, Baltimore, Maryland, p. 28–29.
5. Mutchnick, M. C., Lerner, E. and Conn, H. O. (1974) Portal-systemic encephalopathy and portacaval anastomosis: a prospective controlled investigation. *Gastroenterology*, **66**, 1005–1019
6. LeVeen, H. H. (1985) The LeVeen shunt. *Ann. Rev. Med.*, **36**, 453–469.
7. Campbell, D. P., Parker, D. E. and Anagnostopoulos, C. E. (1973) Survival prediction in portacaval shunt: a computerized statistical analysis. *Am. J. Surg.*, **126**, 748–751.
8. Orloff, M. J., Bell, R. H. Jr and Greenburg, A. G. (1986) Prospective randomized trial of emergency portacaval shunt and medical therapy in unselected cirrhotic patients with bleeding varices. *Gastroenterology*, **90**, 1754 (Abstract).
9. Webster Third New International Dictionary, 1966.
10. Progression of World Best Performances and IAAF Approved World Records, Eds. Wigley, J. and Pearce, J., Peckham, V. Co-ordinated by LOC of IAAF 3rh World Championships in Athletics, 1992.

Harold O Conn
Yale University School of Medicine
New Haven
Connecticut and D.V.A. Medical Center
West Haven, Connecticut 06516, U.S.A.

### THE ROLE OF THE DISTAL SPLENORENAL SHUNT IN THE SCLEROTHERAPY ERA?

### ABSTRACT

*Rikkers, L F., Jin, G., Burnett, D. A., Buchi, K. N. and Cormier, R. A. (1993) Shunt surgery versus endoscopic sclerotherapy for variceal hemorrhage: Late results of a radomized trial. The American Journal of Surgery, 165, 27–33.*

